# Proliferative and Glycolytic Assessment of the Whole-Body Bone Marrow Compartment

**DOI:** 10.4274/mirt.22931

**Published:** 2015-06-17

**Authors:** Mohammed Goryawala, Malek Adjoua, Seza Güleç

**Affiliations:** 1 Miami University Faculty of Medicine, Department of Radiology, Miami, USA; 2 Florida International University Faculty of Medicine, Department of Electrical and Computer Engineering, Miami, USA; 3 Florida International University Herbert Wertheim College, Department of Surgery, Miami, USA

**Keywords:** PET/CT, 18F-FLT, 18F-FDG, bone marrow imaging, image processing

## Abstract

**Objective:**

Quantitative assessment of active bone marrow (BM) in vivo is yet to be well-defined. This study aims to compare total body BM volume estimations obtained from use of both18F-FLT PET/CT and 18F-FDG PET/CT in order to consolidate higher cellular proliferation rates with imaging the highly active red BM in pancreatic cancer.

**Methods:**

This phase I pilot study includes seven patients with pancreatic cancers who underwent both 18F-FLT and 18F-FDG imaging each acquired within a week’s duration. A CT-based classifier is used for segmenting bone into cortical and trabecular regions. The total BM volume is determined through statistical thresholding on PET activity found within the trabecular bone.

**Results:**

Results showed that 18F-FLT measures of red BM volume (RBV) were higher than those obtained from 18F-FDG (∆=89.21 ml). RBV obtained using 18F-FLT in males were found to have high correlation with measured weight (R2=0.61) and BMI (R2=0.70). The red BM fraction obtained from 18F-FLT was significantly different between males and females, with females showing much higher red bone matter within the trabecular bone (p<0.05). In contrast to 18F-FLT, 18F-FDG BM measurements showed that RBV was significantly different between males and females (p<0.05). Results also show that spinal activity SUV threshold for red BM segmentation is significantly different between 18F-FLT PET and 18F-FDG PET (p<0.05).

**Conclusion:**

By combining 18F-FLT-PET and 18F-FDG-PET, this study provides useful insights for in vivo BM estimation through its proliferative and glycolytic activities.

## Abstract (tr)

**Amaç:**

In vivo aktif Kemik İliği’nin (Kİ) kantitatif değerlendirmesi tam olarak tanımlanmış değildir. Bu çalışmadaki amaç 18F-FLT ve 18F FDG görüntüleme yöntemleriyle kemik iliğinin hücresel bölünme aktivitesi ile glikolitik aktivite ile tanımlanan hacimlerinin mukayesesini yapmaktır. Bu spesifik çalışmaya esas olan ana çalışma, pancreas kanserli hastalarda başlatılmış bir faz 1 araştırmasıdır.

**Yöntem:**

By faz 1 çalışmaya pancreas kanseri olan yedi hasta dahil edilmiştir, bir hafta içerisinde hem 18F-FLT hem 18F-FDG görüntülemesi yapılmış, kemik dokusunu kortikal ve trabeküler bölgelere ayırmak için BT bazlı bir sınıflama kullanılmıştır. Total Kİ hacmi, trabeküler kemikteki PET aktivitesinin istatiki bir eşikleme metodu uygulanarak belirlenmiştir.

**Bulgular:**

18F-FLT ile elde edilen kırmızı Kİ hacmi (KKİ) 18F-FDG ile saptanadan daha yüksek bulunmuştur (∆=89,21 ml). 18F-FLT ile saptanan KKİ erkeklerde ağırlık (R2=0,61) ve KKİ (R2=0,70) ile yüksek korelasyon göstermektedir. 18F-FLT ile elde edilen kırmızı Kİ fraksiyonu erkekler ve kadınlar arasında anlamlı farklılık göstermekteydi, kadınlarda trabeküler kemik içerisinde daha yüksek kırmızı kemik maddesi bulunmaktaydı (p<0,05). 18F-FLT’nin aksine 18F-FDG Kİ ölçümleri KKİ’nin kadınlar ve erkekler arsında anlamlı farklılık gösterdiği saptandı (p<0,05). Sonuçlar kırmızı Kİ segmentasyonu için spinal aktivite SUV eşiğinin 18F-FLT PET ile 18F-FDG PET arasında anlamlı derecede farklı olduğunu gösterdi (p<0,05).

**Sonuç:**

Bu çalışma 18F-FLT-PET ile 18F-FDG-PET’in birleştirilmesinin proliferative ve glikolitik aktivitelerine bağlı olarak in vivo Kİ tahminine önemli katkı sağladığını göstermektedir.

## INTRODUCTION

Bone Marrow (BM) is of vital importance in oncological applications since it is most sensitive to radiation and chemotherapy, and often regarded as the dose-limiting factor for systemic radionuclide therapies (1,2,3). Moreover, estimating the volume of the highly active red BM is essential in stem cell transplantation studies (4), as timely stem cell support could improve patient recovery.

Furthermore, clinical studies, which aim to determine the correlation between administered dose and sensitivity to BM toxicity, can be confounded by potential effects of previous treatments.

Despite the advances in the current BM evaluation techniques, biopsy remains the gold standard for clinical diagnosis of functional and morphologic status (5,6,7). However, biopsy that is dependent on site-specific sampling often fails to produce a correct estimation of the whole body BM distribution due to non- homogeneity within the skeletal system.

The International Commission on Radiological Protection (ICRP) estimates that total red marrow, which is the hematopoietically active tissue is 1.170 g in a healthy 40-year-old men and 900 g in women, and a total yellow marrow of about 2.480 g in men, and 1.800 g in women (8,9). Systemic radionuclide therapies mostly affect the red BM, and thus, it is of critical importance to estimate its volume in individuals undergoing treatment. As estimates of BM vary between individuals, patient-specific estimation of hematopoietically active red BM would enable better characterization of the BM compartment and hence better individualized dosimetry (10,11).

Magnetic Resonance Imaging (MRI) based techniques that are capable of estimating water and fat components of the bone may be able to estimate BM volumes (12,13,14). However, the infrequent use and long acquisition times of whole-body MRI hamper their use for BM estimation applications. More recently, quantitative SPECT based techniques using specific radiopharmaceuticals have been developed, which target the reticuloendothelial system as well as erythropoietic or granulopoietic cells for BM estimation (10,15,16). These techniques suffer from the limitation due to tracer kinetics and the physics of single photon detection in SPECT. Recently, a study by Sambuceti et al. estimated the BM volume using 2-deoxy-2-(18F)-fluoro-D-glucose (18F-FDG) PET/CT. The study that was based on a large population is the first of its kind in estimating intraosseous volume (IBV) by using SUV measures obtained from glucose uptake (17). Since glucose consumption in the IBV is indirectly correlated to the glycolytic activity of the BM, the distribution of the FDG PET/CT activity serves as a potent marker for estimating the red marrow within the skeleton.

Prior to 18F-FLT, developed in the 1990s (18,19), a number of markers have been used to predict the biologic behavior of tumors and outcome following surgical and medical treatments. At a basic histological level, mitotic index and lymphovascular invasion are commonly used to assess potentially aggressive tumors. Ki-67 is a nuclear antigen only present in the nuclei of proliferating cells, and good correlation between Ki-67 and S-phase indices have been reported by flow cytometry (20). Ki-67 immunohistochemistry (IHC) has also been used to evaluate a tumors’ proliferative activity.

Clinical evaluation and quantification of proliferative activity and tumor invasiveness can be performed by using FLT-positron emission tomography imaging. Similar to its mother drug azidothymidine, 18F-FLT acts as a terminator of the growing DNA chain. Actually, only a small amount of 18F-FLT is accumulated in DNA; rather, it is retained intracellularly after phosphorylation by thymidine kinase 1. F-FLT pathway is similar to the imaging of glucose uptake pathway with 18F-FDG after trapping by hexokinase. Therefore, both compounds reflect accumulation by transport and subsequent activation by the first step in utilization pathways. 18F-FLT does not reflect the whole DNA synthesis, just as 18F-FDG does not reflect the whole glucose use. All clinical studies demonstrate a distinct BM uptake on 18F-FLT images. Although the exact mechanism is still being discussed, red marrow’s precursor cell population with high proliferative activity is considered as the main reason for 18F-FLT localization.

The primary aim of this study is to estimate the distribution of hematopoietic cellular red BM and non-hematopoietic fibroareolar fatty yellow BM based on a combination of 18F-FLT PET and 18F-FDG PET, which both target BM compartment and evaluate the glycolytic and proliferative properties of BM cells, respectively. In this pilot study, a novel technique is proposed to estimate patient specific BM volumes based on whole body 18F-FLT PET and 18F-FDG PET. The whole bone compartment is first separated into cortical bone and trabecular bone by using.

**Figure 1:**
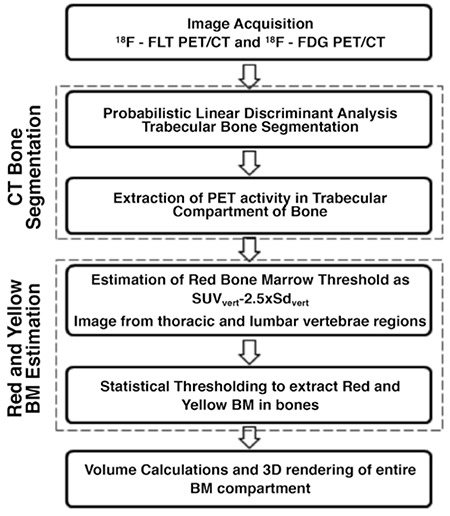
General structure of the entire BM estimation approach, showing the various steps of the algorithm for BM volume estimation

Computed Tomography (CT). The study assumes that the BM resides within the trabecular compartment of the bone and uses statistical thresholds on PET images to estimate the total BM volume within the skeleton. Figure 1 depicts the general structure of BM estimation method.

## MATERIALS AND METHODS

### A. Patient Population and Imaging Protocol

This investigation was performed on patients who underwent 18F-FLT to determine the key imaging characteristics of patients with pancreatic cancer. Eligibility criteria included age ≥18, ability and willingness to give a written consent, life expectancy >3 months, and Karnofsky performance status ≥70. Patient demographics for the studied population are presented in Table 1.

The patient population did not show presence of visible bone metastases on either FDG or FLT PET/CT imaging. In addition, Complete Blood Cell (CBC) counts of the subjects reported an average glucose level of 117.85 mg/dL, a mean RBC count of 4.54x109 cells/ml and a mean platelet count of 385.85x109 per ml. The results of the CBC tests along with absence of any bone metastases rendered the population a viable choice for the study of on BM compartment.

The study was performed under an FDA approved Investigational New Drug (IND) and after IRB review and approval. In this study, 3’-18F-fluoro-3’-deoxy-L- thymidine was obtained from Cardinal Health 414, LLC. 18F- FLT administered activity was 10±1 mCi, and imaging time point was 60±15 minutes post injection, whereas 18F- FDG was administered with an activity of 10±1 mCi, and imaging time point was 60±15 minutes post injection. Images were obtained with 16-slice Siemens PET/CT. The scanning parameters for CT imaging were 140 kVp, 80mA, 0.5s rotation time, and 512×512-pixel matrix.

### B. Probabilistic LD A for Cortical and Trabecular Bone Segmentation

Linear discriminant analysis (LDA) is a widely used technique in pattern recognition, statistics and machine learning to determine characteristic features that can aid in difficult segmentation tasks (21,22,23,24,25). The technique, as used in this study, exploits the fact that Hounsfield values are different in cortical and trabecular bone on CT images (26). This difference in Hounsfield value can be used to train an LDA-based classifier for separating the entire bone into cortical and trabecular compartments.

**Figure 2:**
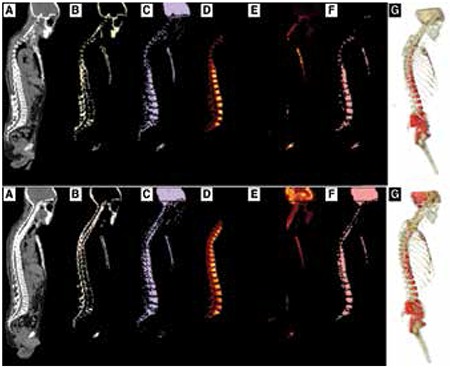
Full body images at various steps of the BM extraction process with the top row showing 18F-FLT and bottom row showing 18F-FDG results. (A) CT image (B) Extracted cortical bone using probabilistic LDA (C) Extracted trabecular bone following probabilistic LDA (D) Activity in trabecular bone in thoracic and lumbar vertebrae regions (E) Activity in trabecular bone in the rest of the body excluding thoracic and lumbar vertebrae regions (F) Thresholded BM map generated after applying a statistical thresholding on PET image. (G) 3D rendering of BM in the entire body, with red regions showing red BM and yellow regions corresponding to yellow BM. The cortical bone overlay is showing using a transparent white overlay

The proposed LDA classifier estimates a posterior probability for each voxel of bone compartment on CT images (Figure 2A), for segmentation of bone into either cortical or trabecular compartments. In order to train the classifier, 1000 voxels of CT skeleton in seven patients are randomly chosen and manually assigned into one of the two groups: Cortical (C) or Trabecular (T).

The training phase estimates the parameters of linear discriminant functions for the two classes as given in Equation 1.

dCL x,y,z=αC+ βC HVx,y,z, and

dTL x,y,z=αT+ βT HV(x,y,z) (1).

αC, βC, αT, βT are the LDA parameters for the two groups cortical (C) and V(x,y,z) represents the Hounsfield value of a particular voxel of the CT image. The pprior=0.5, suggesting that a given voxel of the skeleton has an equal probability of belonging to either one of the two classes.

The segmentation of the entire bone into cortical and trabecular requires each pC or pT based on the linear score LC and LT, respectively. The posterior probabilities signify the likelihood of a voxel to belong to either of the groups. The linear scores LC and LT are estimated based on the Hounsfield value of the voxel under investigation as given by Equation 2.

LC(x,y,z)= dCL x,y,z+log(pprior), and

LTx,y,z= dTL x,y,z+ log(pprior) (2).

The technique computes two 3D posterior probability maps for every voxel of the skeleton. A higher posterior probability determines the grouping of the voxel.

Following this probabilistic segmentation of the skeleton into cortical and trabecular regions, a 3D connected islanding algorithm is employed to remove any spurious points marked erroneously as trabecular bone. The algorithm removes all connected components of the trabecular bone that have fewer than P voxels. The study uses a 26-point neighborhood connectedness for complete 3D connectivity of every voxel that touches the faces, edges, or corners of the voxel under consideration.

The estimation of the trabecular and cortical bone regions is carried out for each patient by CTs co-registered to both the 18F-FLT and 18F-FDG PETs. Figure 2B,C shows the extracted cortical and trabecular regions of bone by using the posterior probability masks generated with Equation 2.

### C. Statistical BM Segmentation

Estimation of hematopoietically active BM is based on the assumption that BM is located in the trabecular compartment of bone, and 18F-FLT voxel intensities, as seen on PET images, are correlated with the proliferative/glycolytic activity of the BM. This estimation technique extracts only the FLT/FDG activity located in the trabecular bone by using the CT-based 3D posterior probability maps obtained in the preceding step. Figure 2E shows the resultant image obtained by masking the registered FLT-PET images with the posterior probability map of the trabecular tissue.

Thresholding in PET images has always been a topic of great interest, with thresholds playing a critical role in determining the correct volume/area of the extracted object (27). Various studies with 18F-FLT have shown different Standard Uptake Value (SUV) thresholds deemed optimal for determining the functionally active BM (28,29). In addition, studies have shown that the SUV threshold varies for different bones of the body, therefore, no one single SUV threshold could possibly be considered as the gold standard (28).

To overcome the contentious issue of thresholds, a completely statistically driven approach outlined by Sambuceti et al. (17) has been adopted for both 18F-FLT and 18F-FDG based BM estimation. The approach initially estimates the mean (SUV) and standard deviation (SDvert) of all the activity located within the trabecular bone, in the thoracic and lumbar vertebrae regions as shown in Figure 2 (D). The technique estimates the red (active) BM volume by accounting all voxels of the trabecular bone that are above the threshold computed as SUVvert −2.5×SDvert. A unique property for estimating the red BM in this study is in the use of patient-specific thresholds rather than a fixed threshold to account for differences in uptake across patients. Less-active yellow BM is estimated as the fraction of the trabecular bone volume below the red BM threshold Figure 2F illustrates the resultant image after the thresholding operation showing the statistically significant voxels of the BM.

### D. Volume Estimation

Cost-effective third party software called ScanIP™ developed by Simpleware Ltd. based in the United Kingdom is utilized for estimating the volume of the statistically significant BM. The calculated volumes are in milliliters (ml). Figure 2G shows 3D rendering of BM in the entire body with red regions showing red BM and yellow regions showing corresponding yellow BM. The cortical bone is shown using a transparent white overlay.

### E. Statistical Analysis

All results are presented in the form of mean ± standard deviation. Linear regressions as performed in this study make use of the least square technique and report the R2 of the fit and the p-value. All comparative results between 18F-FLT and 18F-FDG based estimations are carried out through paired t-tests. A p-value of <0.05 is considered statistically significant.

### F. Ethics Statement

The research has been approved by the Western Institutional Review Board. A Certificate of approval from the WIRB has been obtained. Each participant provided a written consent to participate in the study as per the requirements and guidelines of the IRB.

## RESULTS

### G. Probabilistic LD A Segmentation

To assess the results of the Probabilistic LDA used for segmenting the bone into cortical and trabecular regions, the leave-one-out cross-validation (LOOCV), which is a trusted model for assessing the performance of classifiers, is used in this study. The classification experiment was implemented 50 times on 50 random training sets of 1000 samples. Table 2 provides the average training and LOOCV testing metrics (accuracy, sensitivity, specificity and precision) of the classification results performed across all the 14 CT scans (seven co-registered to 18F-FLT and seven co-registered to 18F-FDG).

### H. Whole Body BM Volumetric Assessment

Estimated BM volumes for the study patients are illustrated in Tables 3 and 4 using 18F-FLT and 18F-FDG, respectively, showing the average red and yellow BM volumes for each patient with the standard deviation obtained upon executing the algorithm 50 times. The tables also provide the volumes of the trabecular bone region along with the fraction of red BM within the trabecular region.

In addition, it is seen that the estimates of Trabecular Bone Volume (IBV) from the CT images co-registered to either FLT or FDG PET/CT were not significantly different (p=0.49). Additionally, it is seen that 18F-FLT based measures of red BM volume (RBV) were higher than those obtained by 18F-FDG (∆=89.21 ml), but results of a paired t-test demonstrate that they were not significantly different (p=0.55). Consecutively, estimated total body yellow BM volume (YBV) from the two different tracers namely 18F-FLT and 18F-FDG were not significantly different (p=0.112). The red BM fraction estimated from 18F-FLT was higher than that estimated from 18F-FDG but was not statistically different for the 7 patients in the study. (∆=0.08; p=0.25).

### I. Effects of Weight, Height, and BMI on Whole Body BM Volumes

**Figure 3:**
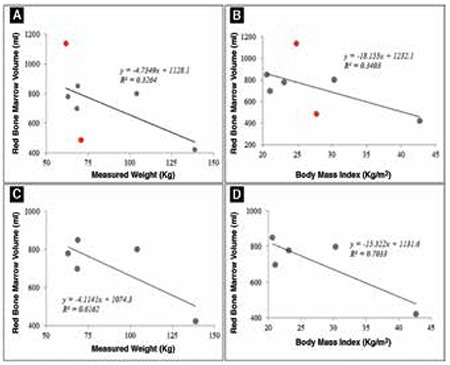
Influence of (A) Measured Body Weight, and (B) Body Mass Index (BMI) on Red BM Volume (RBV) obtained from 18F-FLT imaging in all subjects, whereas (C) and (D) show the influence of measured body weight and BMI in only males, respectively. The RBVs showed better correlation to physical measurements when female subjects were excluded

For 18F-FLT based BM measurements it is seen that although the RBV was not affected by gender (p=0.653), the YBV was significantly different between males and the females (p<0.05). Also, the RBV did not show a good correlation with the measured weight (R2=0.32; Figure 3A), measured height (R2=0.06), Body Mass Index (R2=0.34; Figure 3B) and ideal body weight (R2=0.05). Similar trends were seen for YBV and IBV for the subjects in this study. However, on the exclusion of female subjects (n=2) from regression analysis, it is observed that a higher correlation was obtained for RBV with measured weight (R2=0.61; Figure 3C) and the BMI (R2=0.70; Figure 3D).

**Figure 4:**
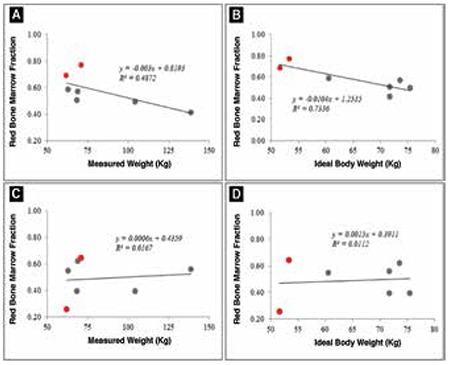
Correlation between Red BM Fraction and (A) Measured Weight and (B) Ideal Body Weight using 18F-FLT PET imaging. Parts (C) and (D) show the same correlation on 18F-FDG PET. A higher correlation was detected between RBV and measured weight and ideal body weight in 18F-FLT based RBV estimations as compared to 18F-FDG imaging

Finally, the red BM fraction was found to be loosely correlated with the measured weight (R2=0.49; Figure 4A) and well correlated to the measured height (R2=0.68) and the ideal body weight (R2=0.73; Figure 4B). It was also significantly different between males and females, with females showing much higher red bone matter within trabecular bone (p<0.05).

For 18F-FDG based BM measurements, it was seen that RBV was significantly different between males and females (p<0.05), however YBV and IBV were not statistically different between the two genders. Also for 18F-FDG, RBV did not correlate well with measured weight (R2=0.24), and Body Mass Index (R2=0.08) and correlated weakly with measured height (R2=0.43) and ideal body weight (R2=0.47).

An interesting feature is seen among males in the study population, where stronger correlation of the IBV with measured weight (R2=0.77), and BMI (R2=0.61) were observed. Also, red BM fraction was not strongly correlated to any demographic features when measured using 18F-FDG PET/CT (Figure 4C and 4D).

### J. SUV Assessment

The SUV measure of 18F-FLT images is an indicator of the proliferative activity of BM, whereas the SUV measure in 18F-FDG imaging is indicative of the glycolytic activity of the BM compartment. Table 5 shows the mean SUV values corresponding to the spinal column and the rest of the body (ROB). Results show that the spinal activity SUV that is used to determine the threshold for red BM segmentation was significantly different between 18F-FLT PET and 18F-FDG PET (p<0.05). Also, the SUV for the rest of the body (excluding the skull) was found to be different between the two imaging tracers. Moreover, the results of an ANOVA for both FDG and FLT based BM estimation suggest that the mean spinal and rest-of-body SUVs were not different among males and females.

**Figure 5:**
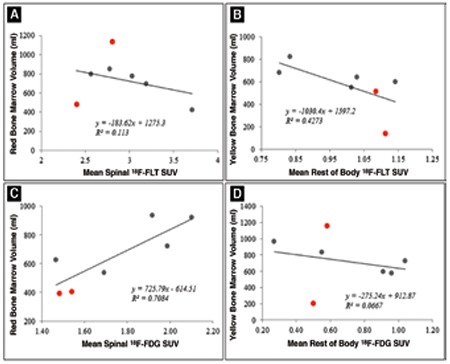
Correlation between (A) Red BM Volume (RBV) to mean spinal SUV and (B) Yellow BM Volume (YBV) to mean rest of body SUV for 18F-FLT imaging. Parts (C) and (D) show the same result for 18F-FDG imaging. RBV are well-correlated for 18F-FDG but not for 18F-FLT imaging

In addition, as seen in Figure 5, red BM volumes were not very well correlated to the mean spinal SUVs for 18F-FLT (R2=0.13; Figure 5A), but were comparatively better correlated in the case of 18F-FDG (R2=0.71; Figure 5C). Also, the yellow BM volumes were not very well correlated with the mean SUVs of the rest of the body for both FLT- and FDG-based estimations (Figure 5B and 5D).

An important finding is that the SUV values in the spine and rest of the body vary significantly for different subjects. This variation is an important aspect that needs to be considered when 18F-FLT images are thresholded using fixed SUV values. This variation in the different statistically meaningful SUV thresholds combined with the fact that the mean SUV value in the trabecular bone varies significantly from patient to patient, suggests that the use of statistical thresholds, like the one suggested in this article, may be advantageous for obtaining more meaningful results.

## DISCUSSION

To the best of our knowledge of the literature, this study is the first to compare total body BM volume estimations obtained from the use of 18F-FLT PET/CT and 18F-FDG PET/CT. The study presented an augmented approach for eliciting a better understanding of the BM compartment by combining the glycolytic activity of 18F-FDG PET and proliferative activity of 18F-FLT PET, each being acquired within a week’s duration for the same patient. Other studies using 18F-FLT for imaging the BM compartment have focused on particular regions of interest rather than providing a measure of the total active BM in the human body (28,30). Also, the nature of the proposed technique renders it possible to extract and quantify the active BM within any bone in the body.

An important contribution relates to the use of the probabilistic LDA technique for extracting compact and trabecular bone compartments. This probabilistic technique is based on the assumption that compact bone in CT images appears as having the highest Hounsfield value and that trabecular bone located in its cavity has a significantly lower Hounsfield value (11,26). This assumption along with the probabilistic training approach enables the technique to be applied on every single slice of the CT image without any human intervention, once the classifier is trained.

Also, as seen in the results, the IBV estimations are not significantly different between those estimated from CTs co-registered to 18F-FLT and 18F-FDG PETs. This finding is expected in such studies since the discrepancies in measurements of the IBV can result in incorrect estimations of BM volumes. Also, since the imaging of FLT and FDG PET/CT is not performed more than a week apart, no expected discrepancies should be observed in the CT-based bone volume measurements as seen in the results. The insignificant differences may be due to the probabilistic nature of the segmentation algorithm for delineating cortical from trabecular bone.

Moreover, the probabilistic LDA based technique classifies the brain as trabecular bone as can be seen in Figure 2C, but it is not a major source of error in BM estimation due to the targeted uptake of 18F-FLT. Brain tissue does not show significant uptake of 18F- FLT, as in the case of FDG, which is the primary reason why the skull needs to be omitted from the quantification purposes. The use of 18F-FLT offers a unique opportunity to quantify the BM volume (albeit negligible) in the skull, which is not estimated in studies based on FDG. It is seen in this study that indeed negligible BM is found in the skull, which is conform to the proposed BM distribution, as was also confirmed in other studies (17,28). The quantitative measurement of the total active BM volume in the body has not been clearly defined in the literature. The primary description of the BM compartment and its related measures come from the 1926 study by Mechanik and its various reviews by Woodward and Holodny, Cristy and Ellis (31,32,33,34).

Mechanik’s study that is based on 13 subjects who died of non-hematological diseases is the fundamental study on which all BM estimates are based (33). A recent study by Sambuceti et al. estimated the BM volume using FDG PET/CT. This study that was based on a large population was first of its kind to estimate intraosseous volume (IBV) using SUV measures obtained from glucose uptake (17).

This study showed that 18F-FLT based BM estimations were higher than those obtained from 18F-FDG PET imaging, all but for two patients. The differences between the BM estimations for the two imaging modalities may be a result of the difference in the distribution and mechanism of the PET tracer within the body.

18F-FLT enables us to visualize the true hematopoietically active BM compartment of the body in order to estimate the whole body volume. Additionally, the assessment of the RBV was very close to the one obtained by Sambuceti et al., which showed that the mean RBV was approximately 541±195 ml.

The measured BM volumes in this study are slightly lower than those reported from other studies, which are based on cadaver studies. There are various reasons that could account for such differences. The difference in values from the Mechanik data can be explained due to the different methods used. Mechanik employed prolonged boiling of the bone in a post-mortem analysis whereas the presented technique involves metabolic imaging.

This study also showed that the RBV volumes estimated by FLT were not significantly different between males and females as was the case using FDG. Other studies based on FDG have showed that assessments of BM were significantly different between the two genders as was confirmed here in this study (17). Also, we found that the mean SUVs of the spinal region and the rest of the body were significantly different between FLT- and FDG-based imaging. The higher mean SUV when using FLT signifies that FLT is a better targeting agent for BM estimation as compared to FDG. Also, the ratio of the mean spinal activity SUV to the mean SUV of the rest of body is higher in FLT imaging (2.92 vs. 2.52 for FDG), which may provide a higher contrast in estimating red BM and hence an improved estimation of RBV.

Although the proposed approach of this study is based on finding statistically meaningful BM volumes, it does have some intrinsic limitations. The primary limiting factor is the effect of partial volume averaging, which is observed both in CT and PET images (35). This may often lead to an overestimation of the cortical and trabecular regions and in turn the BM volumes. Also, motion artifacts due to the long imaging time of the PET as compared to CT is another potential source of estimation error (36). The probabilistic LDA technique has a cross-validated accuracy of 91%, which may be attributed to errors in estimating the boundary between the cortical and trabecular bone regions, which in turn could lead to errors in BM volume measurements. Finally, the size of the population of this pilot study is a primary limitation and current findings are needed to be verified through the inclusion of more subjects as they become available, and as we continue to augment our understanding of pancreatic cancer in order to offer better and more effective means for diagnosis and ultimately better treatment. The small number of patients included in this pilot study is dictated primarily by the nature of the disease which observes the lack of simple early detection methods and the earliest indications of disease being nonspecific. Also, the intensive nature of the imaging task with both 18F-FLT and 18F-FDG PET/CT may have been a limiting factor for inclusion of subjects.

Although some limitations exist, the study provides a unique quantitative assessment of the whole-bone marrow compartment using its proliferative and glycolytic activity in the same patients to provide new insights in the distribution of the active bone marrow for various applications.

## Figures and Tables

**Table 1:**
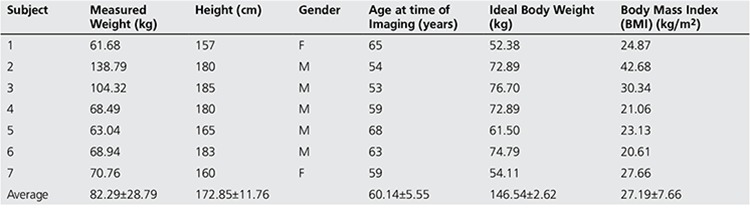
Patient Demographics

**Table 2:**
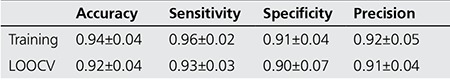
Classification performance

**Table 3:**
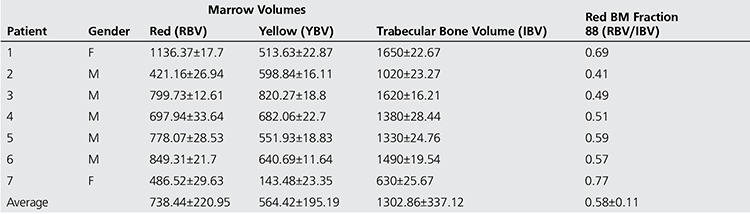
Estimated Whole Body BM volumes using 18F-FLT PET/CT imaging

**Table 4:**
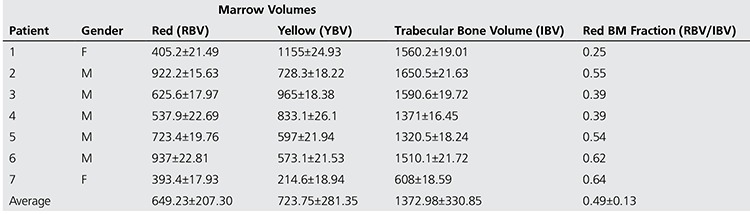
Estimated Whole Body BM volumes using 18F-FDG PET/CT imaging

**Table 5:**
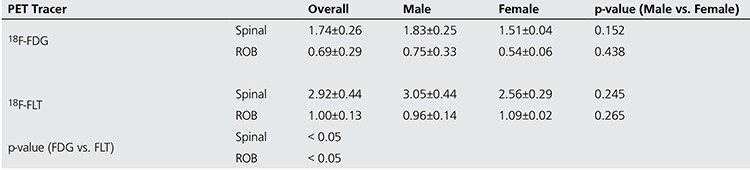
SUV measures
